# Coping With Diagnostic Uncertainty in Antibiotic Prescribing: A Latent Class Study of Primary Care Physicians in Hubei China

**DOI:** 10.3389/fpubh.2021.741345

**Published:** 2021-12-09

**Authors:** Chaojie Liu, Dan Wang, Lixia Duan, Xinping Zhang, Chenxi Liu

**Affiliations:** ^1^School of Psychology and Public Health, La Trobe University, Melbourne, VIC, Australia; ^2^School of Medicine and Health Management, Tongji Medical School, Huazhong University of Science and Technology, Wuhan, China

**Keywords:** diagnostic uncertainty, antibiotic use, physician, primary care, latent class analysis, China

## Abstract

**Background:** Misuse of antibiotics is prevalent worldwide and primary care is a major contributor. Although a clear diagnosis is fundamental for rational antibiotic use, primary care physicians often struggle with diagnostic uncertainty. However, we know little about how physicians cope with this situation and its association with antibiotic prescribing.

**Methods:** A total of 583 primary care physicians were surveyed using the Dealing with Uncertainty Questionnaire. Their prescriptions (*n* = 949,181) over the year 2018 were retrieved retrospectively. Two categories of behavioral patterns of participants were identified based on latent class analyses (high vs. low openness and collaborativeness) in responding to diagnostic uncertainty. Multi-level logistic regression models were established to determine the associations between these behavioral patterns and antibiotic prescribing (overall and broad-spectrum antibiotics) for illness without an indication for antibiotics and those with a conditional indication for antibiotics, respectively, after adjustment for variations of patient (level one) and physician (level two) characteristics.

**Results:** Most physicians reported open communications with their patients (80.96%), collected further information (85.08%), and referred patients to specialists (68.95%) in dealing with diagnostic uncertainly. More than half (56.95%) sought help from colleagues. Less than 20% acted on intuition or adopted a “wait and see” strategy. About 40% participants (*n* = 238) were classified into the group of low openness and collaborativeness in coping with diagnostic uncertainty. They were more likely to prescribe antibiotics for the recorded illness without an indication for antibiotics (AOR = 1.013 for all antibiotics, *p* = 0.024; AOR = 1.047 for broad-spectrum antibiotics, *p* < 0.001), as well as for the recorded illness with a conditional indication for antibiotics (AOR = 1.226 for all antibiotic, *p* < 0.001; AOR = 1.257 for broad-spectrum antibiotics, *p* < 0.001).

**Conclusion:** Low tolerance with diagnostic uncertainty is evident in primary care. Inappropriate and over antibiotic prescribing is shaped by physicians' coping methods of diagnostic uncertainty. Physicians' openness and collaborativeness in responding to diagnostic uncertainty is associated with lower antibiotic prescribing in primary care. Interventions targeting on better management of diagnostic uncertainty may offer a promising approach in reducing antibiotic use in primary care.

## Introduction

Antimicrobial resistance (AMR), a major threat to global health and economic development, has been increasingly recognized as a priority in public health interventions worldwide (1). There is a consensus that the development of AMR has been accelerating at a speed beyond its natural occurrence. This has been fueled, at least partly, by the over- and irrational use of antibiotics (2). Primary care services are widely accessible and have become a major contributor to the over- and irrational use of antibiotics worldwide (2, 3).

The underlying reasons for the over- and irrational use of antibiotics in primary care are complex (4, 5). Diagnostic uncertainty of pathogens has been highlighted as one resulting from a combination of multiple factors, including the early presentation of illness and insufficient diagnostic capacity in primary care settings (6). This represents an inherent nature of primary care. Primary care physicians have to learnt to live with it (7), and prescribe antibiotics in a responsible way under diagnostic uncertainty.

Previous studies have shown that clinical decisions can be shaped by the ability of a physician to manage uncertainty, which subsequently affect patient outcomes (8). A variety of coping strategies in relation to diagnostic uncertainty have been developed in primary care (7), such as shared decision making and referral to specialists. These strategies are believed to have certain benefits for patient care (9). However, there is a lack of empirical evidence to demonstrate their effectiveness in reducing antibiotic prescribing.

China is the biggest consumer of antibiotics in the world (10). Excessive use of antibiotics has been evident in the primary care sector in China. Over half of outpatient visits in China end up with an antibiotic prescription (3), more than 60% of which are deemed inappropriate (11). Existing studies have shown that physician irrational use of antibiotics in primary care in China was associated with limited knowledge (12, 13), mis-attitudes toward antibiotic prescribing (14), patient pressure (15), financial incentives (12, 16) and location of healthcare facilities (15). However, diagnostic uncertainty is a common challenge faced by primary care workers in China (17), which has contributed significantly to the excessive use of antibiotics, in particular in rural facilities (18). Thus, the current study aims to determine the association between coping strategies for diagnostic uncertainty and antibiotic prescribing in primary care in China.

## Participants and Methods

### Setting

This study was conducted in Hubei province in central China, which has a population of over 59 million (4% of all populations in China). According to the World Bank, Hubei is deemed an upper-middle development zone, with its per capita Gross Domestic Product (GDP) reaching US$11,218 in 2019 (19). On average, 334 million annual outpatient visits were recorded in Hubei over the past decade and over half ended with primary care. Primary care services in Hubei are mainly provided by community health services (1,149 registered urban community health centers and 1,129 registered rural township health centers in 2019) (19).

According to the criteria published by the Chinese government, community health services and township health centers always have their own laboratory, with ability to provide at least several basic diagnostic services, including routine blood tests, urine tests, and electrocardiography (20, 21). A recent study showed that roughly 95% of primary care facilities nationwide are able to provide the above diagnostic services (22). The diagnostic services are not free of charge and patients have to pay for the testing. Since China has established a universal health insurance system that covers 97% of its populations, the out of pocket fee is limited (40–60% reimburse rate).

In terms of regulatory measures of antibiotic prescribing, each primary care facility has a committee of rational use of medicine, which is consisted of physicians and administrators. The committee is responsible for evaluating the appropriateness of prescribing based on a randomly selected sample of prescriptions monthly. The results would be used for performance assessment of physicians (23). However, over- and irrational use of antibiotics is prevalent in primary care in Hubei: more than 40% of outpatient visits are prescribed with antibiotics (24), exceeding the benchmark (<30%) recommended by the World Health Organization (15).

### Study Design and Hypotheses

A cross-sectional design was adopted in this study. Coping strategies for diagnostic uncertainty endorsed by the primary care physicians were investigated through a questionnaire survey first. The prescriptions issued by the participating physicians over the past year (2018) were then retrieved retrospectively for data analyses. We classified the primary care physicians into different groups in line with their behavioral patterns in dealing with diagnostic uncertainty and compared their antibiotic prescriptions.

According to Chua et al. (25), certain illness conditions “always” warrant antibiotics, while others “sometimes” or “never” justify antibiotics. In this study, we excluded the cases that “always” warrant antibiotics due to its relatively small sample size and unreliable modeling results. In theory, the cases that “always” warrant antibiotics offer little value for studies focusing on over-use of antibiotics.

The conditions that “never” justify antibiotics cover prescriptions with diagnoses that are unlikely to be caused by bacteria for which antibiotics should not be prescribed (25), for example, upper respiratory tract infections (URTIs). The conditions that “sometimes” justify antibiotics cover prescriptions with diagnoses that antibiotic prescriptions may be needed conditional to a cause of bacterial infections (25), for example, acute tonsillitis.

Previous studies indicate that the effect of diagnostic uncertainty on antibiotic prescribing is most profound for illness with a conditional indication for antibiotics as it leaves a greater space for physician discretion (26, 27). Therefore, we tested two hypotheses in the current study:

- Primary care physicians with different behavioral patterns in dealing with diagnostic uncertainty act differently in antibiotic prescribing;
- The effect of coping strategies for diagnostic uncertainty on antibiotic prescribing is weaker for illness without an indication for antibiotics compared with those with a conditional indication for antibiotics.

### Sampling and Data Collection

A stratified cluster sampling strategy was used in selecting study participants. Five of the 16 municipalities in Hubei, including the capital Wuhan, were purposely identified considering a balance of their geographic location (eastern, central and western) and economic development (high, middle and low). One rural and one urban district were randomly selected from each municipality. All primary care institutions from the selected districts participated in this study. This resulted in a sample of 99 primary care institutions.

Physicians from the participating primary care institutions were invited to complete a questionnaire survey over the period from November 2019 to January 2020 (prior to the outbreak of COVID-19). A total of 764 primary care physicians were approached by the trained investigators and 583 (76.31%) returned valid responses. The prescriptions (*n* = 1,171,921) issued by those who completed the survey over the year of 2018 were retrieved from the records of the local governments retrospectively. The prescription records were anonymised, but contained information in relation to the illness conditions for which the medicines were prescribed and age and gender of the patients. For each prescription, all medications were included and one prescription is for a single episode of a patient. Details about the data collection protocol have been published elsewhere (17).

**Figure 1:**
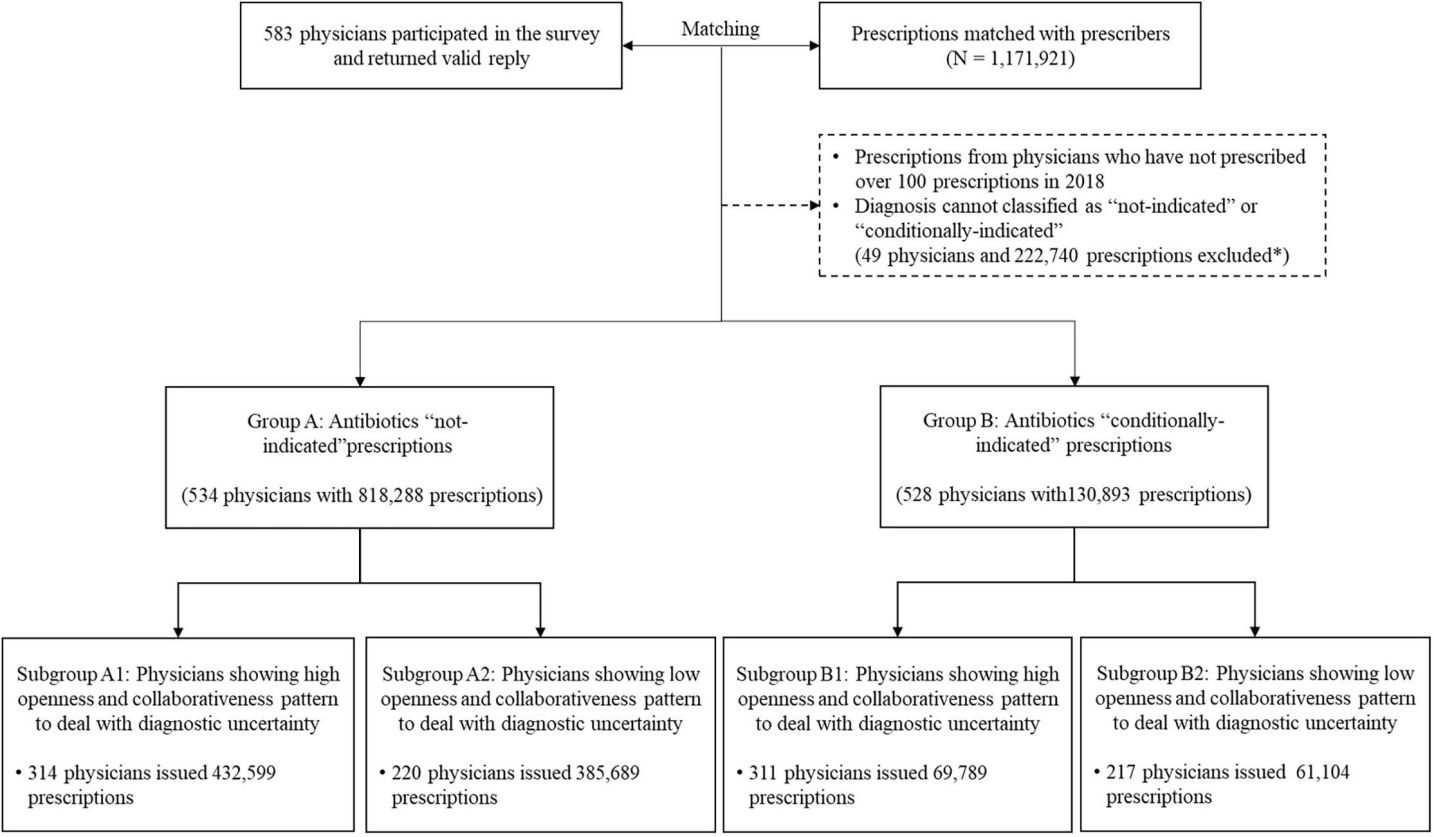
Matching process and characteristics of prescriptions in different groups. Participating physicians and their prescriptions over 2018 were first matched. Based on recorded diagnoses, prescriptions were divided into two groups, namely, illness without an indication for antibiotics (Group A) and illness with an indication conditional for antibiotics (Group B). The former group represents diagnoses that are unlikely to be caused by bacteria for which antibiotics should not be prescribed and the latter one covers diagnoses that antibiotic prescriptions may be needed conditional to a cause of bacterial infections, for example, acute tonsillitis. In either group, two sub-groups were further identified based on physicians' behavioral patterns to cope with diagnostic uncertainty. *Among all the excluded prescriptions, 70.93% prescriptions were excluded due to missing diagnosis, 18.70% were prescriptions with diagnosis requiring antibiotics and 10.37% were due to ineligible physicians (<100 prescriptions during 2018 or missing data of personal characteristics).

To ensure a reliable estimation of antibiotic prescribing, the physicians who prescribed <100 prescriptions in 2018 were excluded (*n* = 49). In addition, the diagnoses that “always” warrant antibiotics were also excluded. This resulted in a final sample of 949,181 prescription records: 818,288 from 534 physicians for illness without an indication for antibiotics and 130,893 from 528 physicians for illness with a conditional indication for antibiotics (Figure 1).

### Measures

#### Dependent Variable

Two outcome indicators were measured: (1) whether a prescription contained systemic use of antibiotics (0 = no and 1 = yes); and (2) whether a prescription contained systemic use of broad-spectrum antibiotics (0 = no and 1 = yes). The systemic use of antibiotics was determined based on the Anatomical Therapeutic and Chemical classification system (coded as J01). Broad-spectrum antibiotics were identified using the criteria of the US national survey (28).

#### Independent Variables

Behavioral patterns of the primary care physicians in dealing with diagnostic uncertainty were the major concern of this study. The study participants were asked about the likelihood of adopting seven different strategies in response to diagnostic uncertainty measured by the Dealing with Uncertainty Questionnaire (DUQ) (7, 29, 30), namely communicating with patients, seeking help from colleagues, referring patients to hospitals (specialties), collecting further information, ordering additional diagnostic tests, acting on intuition and “wait and see”. Each strategy was rated on a five-point Likert scale ranging from “never” to “always”.

According to recent systematic reviews (4, 5), antibiotic prescribing patterns vary by the characteristics of both physicians and patients. In this study, data in relation to the sociodemographic characteristics of the participating physicians (age, gender, educational attainment, annual household income) and their professional experience (years of practice, professional title, sub-specialty, workplace and antibiotic training) were collected. They served as control variables along with the demographic characteristics (age and gender) of the patients.

### Statistical Analysis

Latent class analyses (LCA) were performed to categorize the behavioral patterns adopted by the study participants in dealing with diagnostic uncertainty. The DUQ item responses were collapsed into dichotomous, with 1 indicating “always and often” and 0 indicating “neutral, rarely and never”. We tested the option of one, two, three, four, and five patterns, and selected the best fit model based on the Bayesian information criterion (BIC) and sample adjusted Bayesian information criterion (SABIC), Vuong-Lo-Mendell-Rubin adjusted likelihood ratio test (VLMR-LRT), bootstrapped likelihood ratio test (BLRT), Bayes factor, correct model probability (cmP), and average posterior probability (AvePP) (31). Each study participant was classified into one exclusive group according to the best fit model, indicating a consistent and distinctive pattern of behaviors within the group that differed from others.

The antibiotic prescribing (outcome) indicators among the different LCA groups of physicians were compared for the illness conditions without an indication for antibiotics and the illness conditions with a conditional indication for antibiotics, respectively. Multi-level logistic regression models were established to determine the associations between the LCA grouping and antibiotic prescribing after adjustment for variations in the characteristics of the patients (level one) and the physicians (level two). An enter approach was adopted in the modeling.

We performed sensitivity tests by excluding the recorded illness conditions that attracted low volumes of prescriptions: the bottom 5% or 10% of the volume of prescriptions in each model. We also tested the modeling by excluding the physicians who prescribed <100 prescriptions in each model. In addition, we tested the regression models by replacing the LCA grouping with a split of the study participants 50/50 or 40/60 according to the ranked order of their summed DUQ scores.

The analysis was conducted using STATA (version 12.0) and Mplus (version 6.0). A two-tailed *p*-value of <0.05 was considered as statistically significant.

## Results

### Characteristics of Study Participants

Most of the participating physicians were male (64.32%), aged between 40 and 59 years (64.67%), and worked in rural township health centers (67.75%). Slightly more than half were registered as assistant physicians. About 48% identified themselves as general practitioners. Over 82% indicated that they attended antibiotic training programs (Table 1).

**Table 1:** Characteristics of physician respondents (*n* = 583).

**Characteristics**	***N*** **%**	
Age (years)		
<40	184	31.56%
40–59	377	64.67%
≥60	22	3.77%
Gender		
Male	375	64.32%
Female	208	35.68%
Educational attainment		
Vocational diploma	104	17.84%
Associate medical degree	236	40.48%
Medical degree	243	41.68%
Annual household income (Chinse Yuan ¥)		
<40,000	120	20.58%
40,000–79,999	268	45.97%
80,000–119,999	131	22.47%
≥120,000	64	10.98%
Professional title		
Assistant physician	295	50.60%
Attending physician	220	37.74%
Senior consultant	68	11.66%
Years of clinical experience		
<10	166	28.47%
10–19	181	31.05%
20–29	181	31.05%
≥30	55	9.43%
Workplace		
Urban community health center	188	32.25%
Rural township health center	395	67.75%
Sub-specialty		
General practitioner	282	48.37%
Internist	132	22.64%
Surgeon	67	11.49%
Others (e.g., Pediatrician, Gynecologist)	102	17.50%
Antibiotic training		
Yes	481	82.50%
No	102	17.50%

The study participants prescribed 1,171,921 prescriptions in 2018 and 949,181 (81%) were eligible for inclusion in this study. The patients receiving these prescriptions were predominantly male (51.17%) and 40 years or older (40.36% for 40–64 years; 26.72% for 65 years or older).

### Behavioral Responses to Diagnostic Uncertainty

In dealing with diagnostic uncertainty, most study participants communicated with their patients (80.96%), collected further information (85.08%), and referred their patients to hospital specialists (68.95%). More than half sought help from colleagues (56.95%). However, <40% ordered more diagnostic tests. Only a small percentage acted on intuition or first impression (18.87%) or took a “wait and see” (11.32%) strategy (Table 2).

**Table 2:** Strategies adopted by study participants (*n* = 583) in response to diagnostic uncertainty.

**Coping strategy**	***N*** **%**	
Collecting further information	496	85.08
Communication with patients	472	80.96
Referring patients to hospitals (specialists)	402	68.95
Seeking help from colleagues	332	56.95
Ordering more diagnostic tests	232	39.79
Acting on intuition or first impression	110	18.87
Adopting a “wait and see” strategy	66	11.32

The LCA revealed a best fit model of two distinctive groups of physicians, indicated by several model fit indexes: lowest BIC and SABIC values; highest cmP value; *p* < 0.05 in VLMR-LRT and BLRT (Supplementary Table S1). The best fit model had a high accuracy of classification according to Nylund-Gibson and Choi (31) (AveP*P* > 0.80). One group comprised a higher percentage of physicians endorsing the seven coping behavioral responses compared with the other group, showing relatively higher openness and collaborativeness in dealing with diagnostic uncertainty (Figure 1). The behavioral differences between the two groups were statistically significant in all of the seven coping strategies (*p* < 0.001, Supplementary Table S2). The largest gap appeared in “seeking help from colleagues” (85.51% vs. 15.55%) (Figure 1).

**Figure 2:**
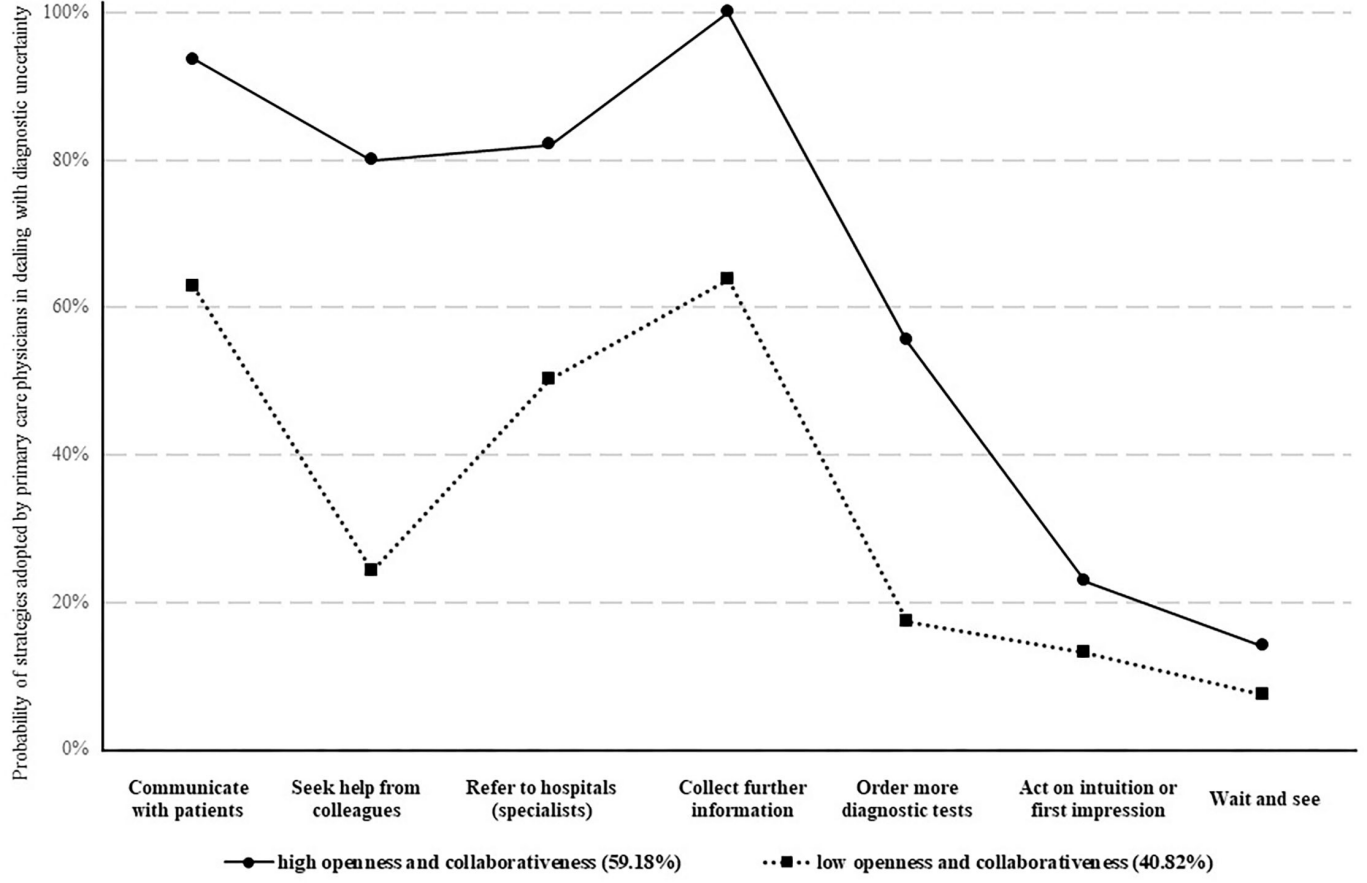
Behavioral patterns of primary care physicians in dealing with diagnostic uncertainty. Two different behavioral patterns of physicians' coping strategies of diagnostic uncertainty were identified. Whether a physician adopted a high or a low openness and collaborativeness to cope with diagnostic uncertainty was classified based on to what likelihood the physician would use the seven approaches to deal with diagnostic uncertainty (presented as different lines). The likelihood of adopting different approaches in coping with diagnostic uncertainty were shown in dots.

About 60% of the participants (*n* = 345) fell into the LCA group of high openness and collaborativeness (Figure 2). Those who were male, younger, less experienced, worked in a rural facility, and attended antibiotic training were more likely to be classified with high openness and collaborativeness (*p* < 0.05, Supplementary Table S2). The split of participants according to their summed DUQ scores, whether 50/50 or 40/60, generated a result of 83% agreement with the LCA classification (*p* < 0.001 in Kappa tests).

### Antibiotic Prescribing and Its Association With Behavioral Responses to Diagnostic Uncertainty

On average, each participating physician issued 1,777 (standard deviation, SD = 2,015) prescriptions, with 43.79% containing antibiotics (32.18% for broad spectrum antibiotics). About 40% of prescriptions for the illness conditions without an indication for antibiotics contained antibiotics (28.93% for broad-spectrum antibiotics). In contrast, 67.54% of prescriptions for the illness conditions with a conditional indication for antibiotics contained antibiotics (52.50% for broad-spectrum antibiotics). Antibiotic prescribing rates differed significantly between the two LCA groups (Supplementary Table S3).

The multi-level logistic regression models showed that the physicians in the group of low openness and collaborativeness were more likely to prescribe antibiotics (adjusted odd ratio, AOR: 1.013, *p* = 0.024) and broad-spectrum antibiotics (AOR: 1.047, *p* < 0.001) for illness without an indication for antibiotics after adjustment for variations in other variables. Such an association was even stronger for illness with a conditional indication for antibiotics, with the physicians in the group of low openness and collaborativenss having an AOR of 1.226 (*p* < 0.001) in prescribing antibiotics (AOR = 1.257 for broad-spectrum antibiotic use, *p* < 0.001) compared with their counterparts in the group of high openness and collaborativeness (Table 3).

**Table 3:** Factors associated with antibiotic prescribing—results of multilevel logistic regression modeling.

**Predictor**	**Illness without an indication for antibiotics**		**Illness with a conditional indication for antibiotics**	
	**AOR for all antibiotics**	**AOR for broad-spectrum antibiotics**	**AOR for all antibiotics**	**AOR for broad-spectrum antibiotics**
**Physicians (level two)**				
**Openness and collaborativeness in responding to diagnostic uncertainty**				
Low (vs high)	1.013 (1.002, 1.024)^*^	1.047 (1.035, 1.059)^***^	1.226 (1.117, 1.345)^***^	1.257 (1.118, 1.414)^***^
Age group	1.012 (0.998, 1.026)	0.973 (0.959, 0.988)^***^	1.064 (0.930, 1.218)	1.484 (1.270, 1.733)^***^
Female gender (vs. male)	0.886 (0.875, 0.898)^***^	0.792 (0.781, 0.803)^***^	0.900 (0.809, 1.000)	0.928 (0.843, 1.021)
Level of education	0.942 (0.934, 0.950)^***^	1.009 (1.000, 1.019)^*^	1.043 (0.927, 1.173)	1.924 (1.741, 2.127)^***^
Household annual income	1.055 (1.048, 1.063)^***^	0.974 (0.967, 0.982)^***^	0.923 (0.871, 0.978)^**^	1.095 (1.031, 1.164)^**^
Professional title	0.994 (0.984, 1.004)	1.009 (0.997, 1.020)	0.674 (0.600, 0.756)^***^	0.630 (0.560, 0.708)^***^
Years of experience	1.000 (0.993, 1.007)	1.004 (0.996, 1.011)	1.117 (1.043, 1.198)^**^	1.058 (0.972, 1.152)
Rural workplace (vs urban)	1.608 (1.583, 1.633)^***^	1.325 (1.303, 1.347)^***^	1.912 (1.630, 2.244)^***^	3.127 (2.657, 3.680)^***^
**Sub-specialty (reference: others)**				
General practitioner	1.244 (1.215, 1.273)^***^	1.196 (1.167, 1.226)^***^	2.341 (1.982, 2.765)^***^	2.803 (2.371, 3.313)^***^
Internalist	1.153 (1.125, 1.181)^***^	1.033 (1.007, 1.060)^*^	1.457 (1.277, 1.663)^***^	0.791 (0.695, 0.901)^***^
Surgeon	1.214 (1.175, 1.253)^***^	0.868 (0.838, 0.899)^***^	1.692 (1.425, 2.009)^***^	1.292 (1.006, 1.660)^*^
Antibiotic training (vs no)	1.176 (1.160, 1.192)^***^	1.339 (1.319, 1.359)^***^	0.955 (0.840, 1.085)	0.709 (0.597, 0.840)^***^
**Patients (level one)**				
**Age (reference:** ** <18 years)**				
18–39	0.667 (0.656, 0.678)^***^	0.911 (0.699, 0.723)^***^	1.081 (1.034, 1.131)^**^	1.055 (1.012, 1.100)^*^
40–64	0.444 (0.438, 0.450)^***^	0.488 (0.481, 0.495)^***^	0.990 (0.951, 1.031)	1.011 (0.974, 1.048)
≥65	0.278 (0.274, 0.283)^***^	0.313 (0.318, 0.318)^***^	0.856 (0.818, 0.896)^***^	0.895 (0.859, 0.933)^***^
Female gender (vs. male)	0.967 (0.958, 0.976)^***^	1.000 (0.990, 1.010)	0.998 (0.971, 1.025)	1.032 (1.007, 1.059)^*^

* *P < 0.05*;

** *P < 0.01*;

*** *P < 0.001*.

The sensitivity analyses produced consistent results when the illness conditions that attracted low volumes of prescriptions were excluded (Supplementary Tables S4, S5), when the physicians who prescribed <100 prescriptions were excluded in each model (Supplementary Table S6), and when the study participants were split in line with their summed DUQ scores (Supplementary Tables S7, S8).

The general practitioners were more likely to prescribe, and the elderly patients (≥65 years) were less likely to be prescribed with antibiotics for both categories of illness conditions. Overall, however, there was a lack of consistent prescribing patterns in line with the characteristics of physicians and patients. Neither qualification education nor antibiotic training was consistently associated with lower odds of antibiotic prescribing (Table 3).

## Discussion

### Main Findings

This study found that the primary care physicians in Hubei had low tolerance towards diagnostic uncertainty. Less than 20% of the study participants acted on intuition or adopted a “wait and see” strategy. Although most participants made efforts to address the challenge of diagnostic uncertainty, two distinctive groups of responding patterns emerged according to the LCA results. One group of physicians was characterized with high openness and collaborativeness and tended to be more tolerant to uncertainty despite taking more actions in responding to diagnostic uncertainty compared with the other group.

High openness and collaborativeness in dealing with diagnostic uncertainty was found to be associated with lower antibiotic prescribing (including broad-spectrum antibiotics) for both illness without an indication and illness with a conditional indication for antibiotics. However, the overall level of antibiotic prescribing remained high, exceeding the 30% benchmark recommended by the World Health Organization (15). Unfortunately, neither qualification education nor antibiotic training demonstrated a consistent association with lower antibiotic prescribing.

### Strengths and Limitations

This study mapped prescriptions with prescribers. The sample size is large, which enabled us to establish reliable multi-level regression modeling. The multi-level modeling methods addressed the concerns of cluster effects, which are common in health services research (32). We used a well-validated instrument to measure coping strategies adopted by the study participants in responding to diagnostic uncertainty (7, 29, 30), and successfully established the link between coping strategies for diagnostic uncertainty and antibiotic prescribing. There is a paucity in the literature documenting the link between diagnostic uncertainty and actual prescribing data (5). Compared with previous studies, the use of LCA for categorizing participants in this study has some advantages. It is able to capture the overall behavioral pattern of each participant without risking a loss of details of each behavioral item embedded in the measurement instrument. Such an approach also reduces concerns of subjectivity in qualitative measurements (6) and information silos arising from an ungrouped approach (7). The robustness of the aforementioned study design was confirmed through several sensitivity tests.

However, this study also has some limitations. The study adopted a cross-sectional design. Prescription data were retrospectively mapped with prescribers. No causal relationships should be assumed for the research findings. The multi-level modeling was established based on the prescribing data without assessing the appropriateness of prescribing, although they were separated into two illness groups in line with the need for antibiotics. In addition, patient outcome indicators were not available for this study. It is also important to note that this study was conducted in the primary care sector in Hubei, China. Any attempts to generalize the findings should be cautious as significant regional disparities often exist and hospitals usually manage patients with more serious conditions. Existing resources available may also impose a significant impact on the prescribing behaviors of physicians. Finally, patient characteristics would influence physicians' responding methods toward diagnostic uncertainty (17). In the current study, general responding methods were measured. Further distinguishment of different dealing patterns within physicians toward different patients would be helpful to further understand how physicians cope with diagnostic uncertainty and how it influences prescribing behaviors. Future study is warranted.

### Comparison With Existing Literature

Previous studies have demonstrated a link between insufficient diagnostic ability and over-prescription of antibiotics (18). Antibiotics are often prescribed when physicians face diagnostic uncertainty or have insufficient capacity to make a correct diagnosis (6, 18). This study advances our understanding by presenting how physicians resolve diagnostic uncertainty and its effects on antibiotic prescribing. In the study, we expanded the coping strategies of primary care physicians in responding to diagnostic uncertainty to a range of measures including communication with patients, collegial support, patient referral, information gathering, diagnostic tests, as well as action on intuition and “wait and see”.

We found that an open and collaborative approach in addressing the challenge of diagnostic uncertainty is associated with lower antibiotic prescribing. The finding is consistent with the results of previous studies, which revealed that antibiotic prescribing is often a stopgap of the physicians' inability to correctly diagnose disease conditions (6, 18). Physicians may prefer unnecessary antibiotic prescribing rather than engaging in long and difficult conversations with patients (or other methods) to deny antibiotics (4). Furthermore, the awareness of the threat of antimicrobial resistance in primary care physicians is usually low (33). As a result, antibiotic prescribing can be considered as a quick fix (34) to satisfy and release patients (4), especially under the context of tense doctor-patient relationships and heavy workloads (35).

This study confirmed the hypothesis that the effect of coping strategies for diagnostic uncertainty on antibiotic prescribing is weaker for illness without an indication for antibiotics compared with those with a conditional indication for antibiotics. The result is consistent with the findings of studies conducted elsewhere (26, 27). Indeed, for an illness condition that requires the discretion of physicians in antibiotic use, such as those that can be caused by either virus or bacteria, a directive approach can offer little value in resource-restricted settings. Instead, a more consultative (deliberation) approach may be more valuable (26). This study showed that <40% of study participants ordered additional diagnostic tests in responding to diagnostic uncertainty, clearly indicating a restriction of available resources and technologies. A retrospective study in the Netherlands proved that shared decision making can reduce conditional use of antibiotics in primary care (27).

It is worth noting that physicians who were older, more experienced, and had a higher professional title were more likely to be categorized in the group of low openness and collaborativeness in dealing with diagnostic uncertainty than the others. This is perhaps because they are supposed to show higher authority and support others in the hierarchical structure of the medical world rather than the other way around (36). A previous study of hospital doctors conducted in England showed that a set of cultural rules govern antibiotic prescribing within the same facility and new and junior physicians are subject to high pressure to follow authorities and seek peer support (37).

### Implications for Research and Policy

Diagnostic uncertainty is an inevitable feature of primary care, which will not disappear anytime soon (38). Appropriate coping strategies for diagnostic uncertainty may offer a promising measure to reduce over- and irrational use of antibiotics in primary care. Empirical evidence shows that primary care physicians often struggle in coping with diagnostic uncertainty by themselves (7). Based on the findings of the current study, we estimate that an open and collaborative approach in responding to diagnostic uncertainty is associated with a 2.83% and 4.70% reduction in overall antibiotic prescribing and broad-spectrum antibiotic prescribing, respectively.

However, relevant interventions should be highly targeted, focusing on those with low endorsement of openness and collaborativeness in responding to diagnostic uncertainty. The LCA results of this study indicate that most primary care physicians in Hubei have already endorsed high openness and collaborativeness in responding to diagnostic uncertainly, which leaves limited room for further improvement.

Empirical evidence shows that multi-faceted interventions are most effective in reducing antibiotic prescriptions (39). These usually involve improvements of both facility diagnostic capacity and physician behaviors (18). Equally important, if not more, is to address the environmental challenges in a system where perverse financial incentives and patient demands are driving the overuse of antibiotics. Under such a system, antibiotic prescribing can be perceived as a quick and easy solution for the complex challenge of managing diagnostic uncertainty (4, 5).

Professional training programs have to be tailored to the needs of the trainees. This study showed that neither qualification education nor antibiotic training had a clear link with reduced prescriptions of antibiotics in primary care. Training for an open and collaborative approach to managing diagnostic uncertainty may offer a better solution. Such training programs should not stand alone due to the complex challenges of over- and irrational use of antibiotics. They have to be integrated with other antibiotic stewardship programs (23). While a directive decision support strategy may be effective in reducing “unwarranted” antibiotic use, deliberation support (dialogue) should be encouraged to reduce conditional antibiotic use (26, 27).

Advocating for an open and collaborative approach for managing diagnostic uncertainty is not without risk. Such an approach may end up with excessively high levels of patient referral and orders of diagnostic tests, leading to a waste of scarce resources (40). Some researchers have advised an increased use of the “wait and see” strategy. Previous studies showed that watchful waiting, including delayed prescriptions, could significantly reduce antibiotic use in primary care without imposing significant adverse outcomes on the patients with acute cough (41), uncomplicated respiratory infections (42), and children with acute otitis media (43). Such a strategy has not been widely endorsed in China and has large space for potential improvement as indicated in this study. Further studies are needed to gather evidence about its effectiveness in China.

## Conclusion

Primary care physicians in Hubei, China have low tolerance towards diagnostic uncertainty. Various strategies have been adopted in dealing with diagnostic uncertainty. An open and collaborative approach in responding to diagnostic uncertainty is associated with lower antibiotic prescribing. Such a link is stronger for illness with a conditional indication for antibiotics that requires the discretion of physicians in prescribing decisions. Targeting appropriate coping strategies for diagnostic uncertainty may offer a promising approach in reducing antibiotic use in primary care.

## Data Availability Statement

The datasets presented in this article are not readily available because the data of this study are derived from surveyed local institutions and restrictions apply to its availability, which were used under license for the current study, and so are not publicly available. Data are however available from the authors upon reasonable request and with permission of surveyed local institutions and governments. Requests to access the datasets should be directed to liu_chenxi@hust.edu.cn.

## Ethics Statement

This study obtained ethics approval from the Research Ethics Committee of Tongji Medical College, Huazhong University of Science and Technology (No: 2020-S099). The participants provided their written informed consent to participate in this study.

## Author Contributions

CheL conceptualized the project and took part in the collection, interpretation of data, and draft and review of the manuscript. ChaL participated in analysis and interpretation of data and writing and review of the manuscript. DW contributed to the acquisition, analysis and interpretation of data. LD and XZ participated in the cleaning and interpretation of data and review of the manuscript. All authors have substantially contributed to the research and read and approved the final version of the article.

## Funding

This study was funded by Independent Innovation Fund of Huazhong University of Science and Technology (Grant No. 2172019kfyXJJS173) and the National Natural Science Foundation of China (Grant No. 71904053). The funding body played no part in the study design, collection, analysis and interpretation of data, writing of the manuscript or the decision to submit the manuscript for publication.

## Conflict of Interest

The authors declare that the research was conducted in the absence of any commercial or financial relationships that could be construed as a potential conflict of interest.

## Publisher's Note

All claims expressed in this article are solely those of the authors and do not necessarily represent those of their affiliated organizations, or those of the publisher, the editors and the reviewers. Any product that may be evaluated in this article, or claim that may be made by its manufacturer, is not guaranteed or endorsed by the publisher.

## Abbreviations

AMR: Antimicrobial resistance
GDP: Gross Domestic Product
URTIs: upper respiratory tract infections
DUQ: Dealing with Uncertainty Questionnaire
LCA: Latent class analyses
BIC: Bayesian information criterion
SABIC: Sample adjusted Bayesian information criterion
VLMR-LRT: Vuong-Lo-Mendell-Rubin adjusted likelihood ratio test
BLRT: bootstrapped likelihood ratio test
cmP: correct model probability
AvePP: average posterior probability
SD: Standard deviation
AOR: Adjusted odd ratio.
