# Supplementary material for: Coping With Diagnostic Uncertainty in Antibiotic Prescribing: A Latent Class Study of Primary Care Physicians in Hubei China
Source: Front Public Health. 2021 Dec 9;9:741345. doi: 10.3389/fpubh.2021.741345 (PMC8695689; doi:10.3389/fpubh.2021.741345)
Supplement: Supplementary file 1 [file Table_1.DOCX]

Supplementary Material

# Table S1

| **Table S1. Model fit statistics of latent class models with different numbers of classified groups** | | | | | | | | |
| --- | --- | --- | --- | --- | --- | --- | --- | --- |
| **Classification** | **BIC** | **SABIC** | **VLMR-LRT** | **BLRT** | **BF** | **Entropy** | **Proportion of physicians classified into the smallest group** | **AvePP** |
| 1-class | 4382.957 | 4360.734 | / | / | 0.001 | / | 100% | - |
| 2-class | 4248.698 | 4201.079 | <0.001 | <0.001 | 3.705 | 0.567 | 40.80% | >0.842 |
| 3-class | 4274.889 | 4201.872 | 0.1718 | <0.001 | 4.513 | 0.674 | 7.00% | >0.833 |
| 4-class | 4305.029 | 4206.615 | 0.0478 | 0.030 | 6.910 | 0.793 | 2.80% | >0.813 |
| 5-class | 4343.688 | 4219.877 | 0.5926 | 0.667 | / | 0.795 | 2.80% | >0.731 |
| 6-class | Failed in model identification tests | | | | | | | |
| Note: BIC: Bayesian Information Criterion; SABIC: Sample-size adjusted BIC; VLMR-LRT: Vuong-Lo-Mendell-Rubin adjusted likelihood ratio test; BLRT: Bootstrapped likelihood ratio test; BF: Bayes Factor; cmP: correct model probability; AvePP: Average posterior probabilities. | | | | | | | | |

# Table S2

| **Table S2. Characteristics of physicians by latent class groups** | | | | | |
| --- | --- | --- | --- | --- | --- |
| **Characteristics (N, %)** | **Group with high openness and collaborativeness**  **(N=345)** | | **Group with low openness and collaborativeness**  **(N=238)** | | ***p**** |
| Age (years) |  |  |  |  |  |
| <40 | 128 | 37.10% | 56 | 23.53% | <0.001 |
| 40-59 | 212 | 61.45% | 165 | 69.33% |  |
| ≥60 | 5 | 1.45% | 17 | 7.14% |  |
| Gender |  |  |  |  |  |
| Male | 234 | 67.83% | 141 | 59.24% | 0.033 |
| Female | 111 | 32.17% | 97 | 40.76% |  |
| Educational attainment |  |  |  |  |  |
| Vocational diploma | 54 | 15.65% | 50 | 21.01% | 0.224 |
| Associate medical degree | 146 | 42.32% | 90 | 37.82% |  |
| Medical degree | 145 | 42.03% | 98 | 41.18% |  |
| Annual household income (Chinese Yuan ¥) |  |  |  |  |  |
| <40,000 | 67 | 19.42% | 53 | 22.27% | 0.087 |
| 40,000-79,999 | 170 | 49.28% | 98 | 41.18% |  |
| 80,000-119,999 | 78 | 22.61% | 53 | 22.27% |  |
| ≥120,000 | 30 | 8.70% | 34 | 14.29% |  |
| Professional title |  |  |  |  |  |
| Assistant physician | 190 | 55.07% | 105 | 44.12% | 0.020 |
| Attending physician | 122 | 35.36% | 98 | 41.18% |  |
| Senior consultant | 33 | 9.57% | 35 | 14.71% |  |
| Yeas of clinical experience |  |  |  |  |  |
| <10 | 106 | 30.72% | 60 | 25.21% | <0.001 |
| 10-19 | 114 | 33.04% | 67 | 28.15% |  |
| 20-29 | 107 | 31.01% | 74 | 31.09% |  |
| ≥30 | 18 | 5.22% | 37 | 15.55% |  |
| Workplace |  |  |  |  |  |
| Urban community health centre | 96 | 27.83% | 92 | 38.66% | 0.006 |
| Rural township health centre | 249 | 72.17% | 146 | 61.34% |  |
| Sub-specialty |  |  |  |  |  |
| General practitioner | 166 | 48.12% | 116 | 48.74% | 0.283 |
| Internist | 85 | 24.64% | 47 | 19.75% |  |
| Surgeon | 41 | 11.88% | 26 | 10.92% |  |
| Others (eg. Pediatrician, Gynecologist) | 53 | 15.36% | 49 | 20.59% |  |
| Antibiotic training |  |  |  |  |  |
| Yes | 298 | 86.38% | 183 | 76.89% | 0.003 |
| No | 47 | 13.62% | 55 | 23.11% |  |
| **Response to diagnostic uncertainty** | | | | | |
| Communication with patients | 331 | 95.94% | 141 | 59.24% | <0.001 |
| Seeking help from colleagues | 295 | 85.51% | 37 | 15.55% | <0.001 |
| Referring patients to hospitals (specialists) | 283 | 82.03% | 119 | 50.00% | <0.001 |
| Collecting further information | 345 | 100.00% | 151 | 63.45% | <0.001 |
| Ordering additional diagnostic tests | 194 | 56.23% | 38 | 15.97% | <0.001 |
| Acting on intuition or first impression | 84 | 24.35% | 26 | 10.92% | <0.001 |
| Adopting a “wait and see” strategy | 52 | 15.07% | 14 | 5.88% | <0.001 |
| *Chi-square or Fisher exact tests | | | | | |

# Table S3

| **Table S3. Characteristics of prescriptions and patients by latent class groups** | | | | | | | | | | | | | | | |
| --- | --- | --- | --- | --- | --- | --- | --- | --- | --- | --- | --- | --- | --- | --- | --- |
| **Characteristics (N, %)** | **Illness without an indication for antibiotics** | | | | | | |  | **Illness with a conditional indication for antibiotics** | | | | | | |
|  | **Total** | | **Openness and collaborativeness** | | | | ***P**** |  | **Total** | | **Openness and collaborativeness** | | | | ***P**** |
|  |  |  | High | | Low | |  |  |  |  | High | | Low | |  |
| Number of prescriptions | 818,288 | | 432,599 | | 385,689 | |  |  | 130,893 | | 69,789 | | 61,104 | |  |
| Prescriptions containing antibiotics | 327,203 | 39.99% | 177,895 | 41.12% | 149,308 | 38.71% | <0.001 |  | 88,403 | 67.54% | 48,506 | 69.50% | 39,897 | 65.29% | <0.001 |
| Prescriptions containing broad-spectrum antibiotics | 236,753 | 28.93% | 128,272 | 29.65% | 108,481 | 28.13% | <0.001 |  | 68,722 | 52.50% | 38,087 | 54.57% | 30,635 | 50.14% | <0.001 |
| Patient age (years) |  |  |  |  |  |  | <0.001 |  |  |  |  |  |  |  | <0.001 |
| <18 | 132,574 | 16.20% | 79,585 | 18.40% | 52,989 | 13.74% |  |  | 30,244 | 23.11% | 18,681 | 26.77% | 11,563 | 18.92% |  |
| 18-39 | 122,876 | 15.02% | 61,000 | 14.10% | 61,876 | 16.04% |  |  | 26,781 | 20.46% | 13,254 | 18.99% | 13,527 | 22.14% |  |
| 40-64 | 336,051 | 41.07% | 179,925 | 41.59% | 156,126 | 40.48% |  |  | 47,068 | 35.96% | 24,306 | 34.83% | 22,762 | 37.25% |  |
| ≥65 | 226,787 | 27.71% | 112,089 | 25.91% | 114,698 | 29.74% |  |  | 26,800 | 20.47% | 13,548 | 19.41% | 13,252 | 21.69% |  |
| Patient gender |  |  |  |  |  |  | 0.038 |  |  |  |  |  |  |  | 0.187 |
| Male | 418,339 | 51.12% | 220,693 | 51.02% | 197,646 | 51.24% |  |  | 66,228 | 50.60% | 35,192 | 50.43% | 31,036 | 50.79% |  |
| Female | 399,949 | 48.88% | 211,906 | 48.98% | 188,043 | 48.76% |  |  | 64,665 | 49.40% | 34,597 | 49.57% | 30,068 | 49.21% |  |
| *Chi-square or Fisher exact tests | | | | | | | | | | | | | | | |

# Table S4

| **Table S4. Sensitivity tests – multi-level logistic regression modelling on antibiotic prescribing**  **(by excluding the recorded illness conditions that attracted the bottom 5% low volumes of prescriptions in each model)** | | | | |
| --- | --- | --- | --- | --- |
| **Predictor** | **Illness without an indication for antibiotics** | | **Illness with a conditional indication for antibiotics** | |
|  | **AOR for all antibiotics** | **AOR for Broad-spectrum antibiotics** | **AOR for all antibiotics** | **AOR for Broad-spectrum antibiotics** |
| **Physicians (Level Two)** | | | | |
| Openness and collaborativeness in responding to diagnostic uncertainty | | | | |
| Low (vs High) | 1.015 (1.004, 1.026)** | 1.052 (1.039, 1.064)*** | 1.239 (1.127, 1.362)*** | 1.292 (1.147, 1.454)*** |
| Age group | 1.020 (1.006, 1.034)** | 0.976 (0.962, 0.991)** | 1.024 (0.893, 1.175) | 1.483 (1.268, 1.735)*** |
| Female gender (vs male) | 0.870 (0.858, 0.881)*** | 0.781 (0.769, 0.792)*** | 0.858 (0.770, 0.956)** | 0.895 (0.812, 0.986)* |
| Level of education | 0.940 (0.932, 0.949)*** | 1.005 (0.996, 1.015) | 1.040 (0.923, 1.173) | 1.926 (1.741, 2.130)*** |
| Household annual income | 1.058 (1.051, 1.066)*** | 0.979 (0.971, 0.987)*** | 0.917 (0.864, 0.973)** | 1.087 (1.023, 1.156)** |
| Professional title | 0.990 (0.980, 1.001) | 1.004 (0.992, 1.016) | 0.633 (0.563, 0.712)*** | 0.614 (0.546, 0.691)*** |
| Years of experience | 0.999 (0.992, 1.006) | 1.007 (0.999, 1.015) | 1.181 (1.100, 1.267)*** | 1.082 (0.994, 1.179) |
| Rural workplace (vs urban) | 1.597 (1.572, 1.623)*** | 1.317 (1.295, 1.340)*** | 1.851 (1.572, 2.179)*** | 3.001 (2.543, 3.542)*** |
| Sub-specialty (reference: others) | | | | |
| General practitioner | 1.282 (1.252, 1.313)*** | 1.224 (1.194, 1.255)*** | 2.510 (2.114, 2.981)*** | 2.973 (2.504, 3.531)*** |
| Internalist | 1.179 (1.150, 1.208)*** | 1.053 (1.026, 1.080)*** | 1.529 (1.336, 1.749)*** | 0.823 (0.722, 0.938)** |
| Surgeon | 1.233 (1.192, 1.274)*** | 0.863 (0.832, 0.895)*** | 1.733 (1.454, 2.065)*** | 1.377 (1.067, 1.778)* |
| Antibiotic training (vs no) | 1.203 (1.187, 1.220)*** | 1.370 (1.349, 1.391)*** | 0.964 (0.846, 1.098) | 0.729 (0.614, 0.865)***+ |
| **Patients (Level One)** |  |  |  |  |
| Age (reference: <18 years) |  |  |  |  |
| 18-39 | 0.671 (0.660, 0.683)*** | 0.729 (0.707, 0.731)*** | 1.035 (1.019, 1.119)** | 1.042 (0.998, 1.087) |
| 40-64 | 0.430 (0.424, 0.436)*** | 0.477 (0.471, 0.484)*** | 1.036 (0.994, 1.080) | 1.030 (0.992, 1.070) |
| ≥65 | 0.267 (0.263, 0.271)*** | 0.303 (0.298, 0.308)*** | 0.884 (0.844, 0.926)*** | 0.909 (0.871, 0.948)*** |
| Female gender (vs male) | 0.971 (0.962, 0.981)*** | 1.007 (0.996, 1.017) | 1.014 (0.986, 1.042) | 1.042 (1.015, 1.069)** |
| **P*<0.05; ***P*<0.01; ****P*<0.001 | | | | |

# Table S5

| **Table S5: Sensitivity tests – multi-level logistic regression modelling on antibiotic prescribing**  **(by excluding the recorded illness conditions that attracted the bottom 10% low volumes of prescriptions in each model)** | | | | |
| --- | --- | --- | --- | --- |
| **Predictor** | **Illness without an indication for antibiotics** | | **Illness with a conditional indication for antibiotics** | |
|  | **AOR for all antibiotics** | **AOR for broad-spectrum antibiotics** | **AOR for all antibiotics** | **AOR for broad-spectrum antibiotics** |
| **Physicians (Level Two)** | | | | |
| Openness and collaborativeness in responding to diagnostic uncertainty | | | | |
| Low (vs High) | 1.014 (1.003, 1.026)* | 1.048 (1.035, 1.060)*** | 1.144 (1.040, 1.260)** | 1.269 (1.127, 1.430)*** |
| Age group | 1.018 (1.003, 1.032)* | 0.978 (0.963, 0.993)** | 0.912 (0.837, 1.105) | 1.452 (1.240, 1.699)*** |
| Female gender (vs male) | 0.859 (0.847, 0.871)*** | 0.759 (0.748, 0.771)*** | 0.883 (0.792, 0.985)* | 0.891 (0.808, 0.982)* |
| Level of education | 0.952 (0.944, 0.961)*** | 1.013 (1.003, 1.023)** | 1.021 (0.904, 1.153) | 1.962 (1.772, 2.173)*** |
| Household annual income | 1.048 (1.040, 1.056)*** | 0.968 (0.960, 0.976)*** | 0.904 (0.851, 0.959)** | 1.074 (1.009, 1.142)* |
| Professional title | 0.985 (0.974, 0.996)** | 1.007 (0.994, 1.019) | 0.599 (0.532, 0.675)*** | 0.601 (0.534, 0.677)*** |
| Years of experience | 0.998 (0.991, 1.005) | 1.005 (0.997, 1.013) | 1.203 (1.120, 1.292)*** | 1.094 (1.004, 1.192)* |
| Rural workplace (vs urban) | 1.550 (1.525, 1.575)*** | 1.287 (1.264, 1.310)*** | 1.849 (1.568, 2.180)*** | 2.974 (2.517, 3.515)*** |
| Sub-specialty (reference: others) | | | | |
| General practitioner | 1.233 (1.202, 1.264)*** | 2.448 (2.056, 2.913)*** | 3.032 (2.550, 3.605)*** | 1.233 (1.202, 1.264)*** |
| Internalist | 1.076 (1.048, 1.104)*** | 1.501 (1.310, 1.719)*** | 0.825 (0.723, 0.941)** | 1.076 (1.048, 1.104)*** |
| Surgeon | 0.871 (0.839, 0.904)*** | 1.967 (1.644, 2.352)*** | 1.728 (1.327, 2.249)*** | 0.871 (0.839, 0.904)*** |
| Antibiotic training (vs no) | 1.366 (1.345, 1.387)*** | 0.936 (0.820, 1.068) | 0.714 (0.600, 0.849)*** | 1.366 (1.345, 1.387)*** |
| **Patients (Level One)** |  |  |  |  |
| Age (reference: <18 years) |  |  |  |  |
| 18-39 | 0.674 (0.662, 0.686)*** | 0.719 (0.706, 0.731)*** | 1.085 (1.034, 1.139)** | 1.057 (1.011, 1.104)* |
| 40-64 | 0.439 (0.433, 0.446)*** | 0.486 (0.479, 0.493)*** | 1.077 (1.031, 1.125)** | 1.066 (1.025, 1.108)** |
| ≥65 | 0.277 (0.273, 0.281)*** | 0.314 (0.309, 0.319)*** | 0.910 (0.867, 0.955)*** | 0.935 (0.895, 0.977)** |
| Female gender (vs male) | 0.977 (0.967, 0.986)*** | 1.012 (1.001, 1.022)* | 1.016 (0.988, 1.046) | 1.044 (1.016, 1.072)** |
| **P*<0.05; ***P*<0.01; ****P*<0.001 | | | | |

# Table S6

| **Table S6. Sensitivity tests – multi-level logistic regression modelling on antibiotic prescribing**  **(by excluding the physician who prescribed less than 100 prescriptions in each model)** | | | | |
| --- | --- | --- | --- | --- |
| **Predictor** | **Illness without an indication for antibiotics** | | **Illness with a conditional indication for antibiotics** | |
|  | **AOR for all antibiotics** | **AOR for broad-spectrum antibiotics** | **AOR for all antibiotics** | **AOR for broad-spectrum antibiotics** |
| **Physicians (Level Two)** | | | | |
| Openness and collaborativeness in responding to diagnostic uncertainty | | | | |
| Low (vs High) | 1.013 (1.002, 1.024)* | 1.047 (1.035, 1.059)*** | 1.167 (1.049, 1.297)** | 1.258 (1.093, 1.446)** |
| Age group | 1.012 (0.998, 1.026) | 0.973 (0.959, 0.988)*** | 1.084 (0.932, 1.261) | 1.578 (1.314, 1.894)*** |
| Female gender (vs male) | 0.886 (0.875, 0.898)*** | 0.792 (0.781, 0.803)*** | 0.968 (0.862, 1.087) | 1.022 (0.917, 1.138) |
| Level of education | 0.942 (0.934, 0.950)*** | 1.009 (1.000, 1.019)* | 1.035 (0.902, 1.188) | 2.192 (1.953, 2.460)*** |
| Household annual income | 1.055 (1.048, 1.063)*** | 0.974 (0.967, 0.982)*** | 0.916 (0.859, 0.976)** | 1.068 (0.998, 1.143) |
| Professional title | 0.994 (0.983, 1.004) | 1.008 (0.997, 1.020) | 0.646 (0.568, 0.735)*** | 0.605 (0.529, 0.693)*** |
| Years of experience | 1.000 (0.993, 1.007) | 1.004 (0.996, 1.011) | 1.095 (1.015, 1.181)* | 1.004 (0.910, 1.109) |
| Rural workplace (vs urban) | 1.608 (1.583, 1.633)*** | 1.324 (1.302, 1.347)*** | 2.086 (1.726, 2.520)*** | 3.845 (3.150, 4.693)*** |
| Sub-specialty (reference: others) | | | | |
| General practitioner | 1.243 (1.215, 1.273)*** | 1.196 (1.166, 1.225)*** | 2.172 (1.812, 2.604)*** | 2.499 (2.063, 3.028)*** |
| Internalist | 1.152 (1.124, 1.180)*** | 1.033 (1.006, 1.059)* | 1.376 (1.191, 1.589)*** | 0.724 (0.629, 0.832)*** |
| Surgeon | 1.214 (1.175, 1.253)*** | 0.868 (0.838, 0.900)*** | 1.803 (1.476, 2.203)*** | 1.156 (0.856, 1.560) |
| Antibiotic training (vs no) | 1.176 (1.160, 1.192)*** | 1.339 (1.319, 1.359)*** | 0.981 (0.852, 1.128) | 0.654 (0.533, 0.803)*** |
| **Patients (Level One)** |  |  |  |  |
| Age (reference: <18 years) |  |  |  |  |
| 18-39 | 0.667 (0.656, 0.678)*** | 0.711 (0.699, 0.723)*** | 1.059 (1.010, 1.110)* | 1.026 (0.982, 1.071) |
| 40-64 | 0.444 (0.438, 0.450)*** | 0.488 (0.481, 0.495)*** | 0.995 (0.954, 1.038) | 1.006 (0.969, 1.045) |
| ≥65 | 0.278 (0.274, 0.283)*** | 0.313 (0.308, 0.318)*** | 0.855 (0.815, 0.896)*** | 0.892 (0.854, 0.932)*** |
| Female gender (vs male) | 0.967 (0.958, 0.976)*** | 1.000 (0.990, 1.010) | 1.009 (0.981, 1.037) | 1.035 (1.008, 1.062)* |
| **P*<0.05; ***P*<0.01; ****P*<0.001 | | | | |

# Table S7

| **Table S7. Sensitivity tests – multi-level logistic regression modelling on antibiotic prescribing**  **(by splitting physicians into two groups (50% vs. 50%) in line with their summed DUQ scores)** | | | | |
| --- | --- | --- | --- | --- |
| **Predictor** | **Illness without an indication for antibiotics** | | **Illness with a conditional indication for antibiotics** | |
|  | **AOR for all antibiotics** | **AOR for broad-spectrum antibiotics** | **AOR for all antibiotics** | **AOR for broad-spectrum antibiotics** |
| **Physicians (Level Two)** | | | | |
| Openness and collaborativeness in responding to diagnostic uncertainty | | | | |
| Low (vs High) | 1.045 (1.034, 1.056)*** | 1.072 (1.059, 1.084)*** | 1.299 (1.185, 1.424)*** | 1.318 (1.173, 1.481)*** |
| Age group | 1.011 (0.997, 1.025) | 0.973 (0.959, 0.988)*** | 1.075 (0.945, 1.224) | 1.497 (1.292, 1.735)*** |
| Female gender (vs male) | 0.885 (0.873, 0.896)*** | 0.788 (0.777, 0.800)*** | 0.864 (0.775, 0.962)** | 0.868 (0.788, 0.957)** |
| Level of education | 0.943 (0.935, 0.951)*** | 1.010 (1.001, 1.019)* | 0.966 (0.860, 1.084) | 1.776 (1.607, 1.962)*** |
| Household annual income | 1.054 (1.047, 1.062)*** | 0.972 (0.965, 0.980)*** | 0.929 (0.877, 0.984)* | 1.128 (1.060, 1.199)*** |
| Professional title | 1.000 (0.989, 1.010) | 1.015 (1.003, 1.027)* | 0.745 (0.661, 0.839)*** | 0.684 (0.602, 0.777)*** |
| Years of experience | 0.997 (0.990, 1.004) | 0.999 (0.991, 1.006) | 1.068 (0.993, 1.148) | 1.018 (0.931, 1.113) |
| Rural workplace (vs urban) | 1.623 (1.598, 1.649)*** | 1.336 (1.313, 1.358)*** | 1.901 (1.623, 2.227)*** | 3.091 (2.627, 3.636)*** |
| Sub-specialty (reference: others) | | | | |
| General practitioner | 1.241 (1.213, 1.270)*** | 1.197 (1.167, 1.226)*** | 2.410 (2.045, 2.841)*** | 2.811 (2.379, 3.322)*** |
| Internalist | 1.149 (1.122, 1.177)*** | 1.036 (1.010, 1.062)** | 1.455 (1.280, 1.655)*** | 0.778 (0.683, 0.886)*** |
| Surgeon | 1.210 (1.172, 1.250)*** | 0.870 (0.840, 0.901)*** | 1.598 (1.351, 1.891)*** | 1.269 (0.988, 1.630) |
| Antibiotic training (vs no) | 1.180 (1.164, 1.196)*** | 1.341 (1.321, 1.361)*** | 0.939 (0.830, 1.062) | 0.657 (0.572, 0.754)*** |
| **Patients (Level One)** |  |  |  |  |
| Age (reference: <18 years) |  |  |  |  |
| 18-39 | 0.667 (0.655, 0.677)*** | 0.710 (0.698, 0.722)*** | 1.082 (1.034, 1.132)** | 1.055 (1.013, 1.010)* |
| 40-64 | 0.444 (0.438, 0.450)*** | 0.488 (0.481, 0.494)*** | 0.991 (0.982, 1.032) | 1.011 (0.975, 1.049) |
| ≥65 | 0.278 (0.274, 0.282)*** | 0.312 (0.307, 0.317)*** | 0.857 (0.819, 0.896)*** | 0.896 (0.859, 0.933)*** |
| Female gender (vs male) | 0.967 (0.958, 0.976)*** | 0.999 (0.990, 1.009) | 0.997 (0.971, 1.025) | 1.032 (1.007, 1.059)* |
| **P*<0.05; ***P*<0.01; ****P*<0.001 | | | | |

# Table S8

| **Table S8. Sensitivity tests – multi-level logistic regression modelling on antibiotic prescribing**  **(by splitting physicians into two groups (60% vs. 40%) in line with their summed DUQ scores)** | | | | |
| --- | --- | --- | --- | --- |
| **Predictor** | **Illness without an indication for antibiotics** | | **Illness with a conditional indication for antibiotics** | |
|  | **AOR for all antibiotics** | **AOR for broad-spectrum antibiotics** | **AOR for all antibiotics** | **AOR for broad-spectrum antibiotics** |
| **Physicians (Level Two)** | | | | |
| Openness and collaborativeness in responding to diagnostic uncertainty | | | | |
| Low (vs High) | 1.069 (1.055, 1.078)*** | 1.115 (1.102, 1.128)*** | 1.172 (1.087, 1.265)*** | 1.333 (1.214, 1.462)*** |
| Age group | 1.011 (0.998, 1.025) | 0.973 (0.959, 0.987)*** | 1.001 (0.884, 1.133) | 1.457 (1.276, 1.663)*** |
| Female gender (vs male) | 0.880 (0.869, 0.892)*** | 0.781 (0.770, 0.793)*** | 0.920 (0.829, 1.021) | 0.895 (0.814, 0.984)* |
| Level of education | 0.944 (0.936, 0.952)*** | 1.011 (1.002, 1.021)* | 0.973 (0.867, 1.092) | 1.749 (1.583, 1.932)*** |
| Household annual income | 1.059 (1.051, 1.066)*** | 0.979 (0.971, 0.986)*** | 0.922 (0.870, 0.977)** | 1.151 (1.081, 1.224)*** |
| Professional title | 0.998 (0.988, 1.009) | 1.014 (1.002, 1.025)* | 0.723 (0.642, 0.814)*** | 0.691 (0.612, 0.782)*** |
| Years of experience | 0.998 (0.991, 1.005) | 1.001 (0.993, 1.008) | 1.099 (1.024, 1.180)** | 1.021 (0.938, 1.111) |
| Rural workplace (vs urban) | 1.623 (1.598, 1.648)*** | 1.337 (1.315, 1.359)*** | 1.892 (1.615, 2.217)*** | 3.044 (2.588, 3.581)*** |
| Sub-specialty (reference: others) | | | | |
| General practitioner | 1.231 (1.202, 1.260)*** | 1.179 (1.151, 1.209)*** | 2.456 (2.084, 2.894)*** | 2.819 (2.387, 3.329)*** |
| Internalist | 1.140 (1.113, 1.168)*** | 1.021 (0.996, 1.048) | 1.522 (1.342, 1.726)*** | 0.785 (0.691, 0.891)*** |
| Surgeon | 1.192 (1.154, 1.231)*** | 0.849 (0.820, 0.880)*** | 1.822 (1.562, 2.126)*** | 1.284 (1.000, 1.649) |
| Antibiotic training (vs no) | 1.189 (1.173, 1.205)*** | 1.359 (1.339, 1.380)*** | 0.922 (0.815, 1.043) | 0.686 (0.575, 0.748)*** |
| **Patients (Level One)** |  |  |  |  |
| Age (reference: <18 years) |  |  |  |  |
| 18-39 | 0.667 (0.656, 0.678)*** | 0.712 (0.700, 0.7224)*** | 1.081 (1.033, 1.131)** | 1.056 (1.014, 1.101)** |
| 40-64 | 0.444 (0.438, 0.450)*** | 0.489 (0.482, 0.495)*** | 0.990 (0.951, 1.031) | 1.012 (0.976, 1.050) |
| ≥65 | 0.278 (0.274, 0.282)*** | 0.313 (0.308, 0.318)*** | 0.856 (0.818, 0.896)*** | 0.896 (0.860, 0.934)*** |
| Female gender (vs male) | 0.966 (0.957, 0.975)*** | 0.998 (0.988, 1.008) | 0.997 (0.971, 1.025) | 1.032 (1.006, 1.058)* |
| **P*<0.05; ***P*<0.01; ****P*<0.001 | | | | |
